# Ein seltener Fall von lepromatöser Lepra in Deutschland

**DOI:** 10.1111/ddg.15801_g

**Published:** 2025-11-14

**Authors:** Roman Saternus, Gisela Bretzel, Tamaz Tsulaia, Kristin Burckhardt, Krista Yordanova, Sophie Elisabeth Müller, Steffen Jahn, Thomas Vogt, Sophie Schneitler

**Affiliations:** ^1^ Klinik für Dermatologie Venerologie und Allergologie am Universitätsklinikum des Saarlandes, Homburg; ^2^ Institut für Infektions‐ und Tropenmedizin am Klinikum der Universität München; ^3^ Institut für Medizinische Mikrobiologie und Hygiene am Universitätsklinikum des Saarlandes, Homburg; ^4^ Pathologie an der Dresdner Heide, Radeberg

Sehr geehrte Herausgeber,

Ein 56‐jähriger Patient stellte sich erstmals in unserer Hochschulambulanz vor. Nach eigenen Angaben bestanden seit einigen Monaten größenprogrediente, störende Läsionen im Gesicht sowie an den distalen Unterarmen. Er berichtete über verminderte Sensibilität innerhalb der betroffenen Areale. Der Patient stammt ursprünglich aus Ghana und hält sich seit einem längeren, nicht genau zu bestimmenden Zeitraum in Deutschland auf, wo er in der Reinigung eines Hotels tätig ist. Zuvor war er durch niedergelassene dermatologische und HNO‐ärztliche Kollegen unter der Arbeitsdiagnose Rhinophym behandelt worden, jedoch ohne klinische Besserung. Bis auf eine monoklonale IgG‐Gammopathie unklarer Signifikanz sind keine Vorerkrankungen bekannt; eine Dauermedikation besteht nicht.

Es zeigten sich zentrofazial unter Beteiligung von Stirn, Nase, Wange und Lippe symmetrische, hypästhetische, konfluierende hautfarbene Papeln und Noduli ohne epidermale Veränderungen. Es bestand zudem eine Rarefizierung der lateralen Augenbrauen (Abbildung 1). An den distalen Unterarmen imponierten einzelne, ulnarseitig gelegene, subkutane, indolente Nodi.

**ABBILDUNG 1:**
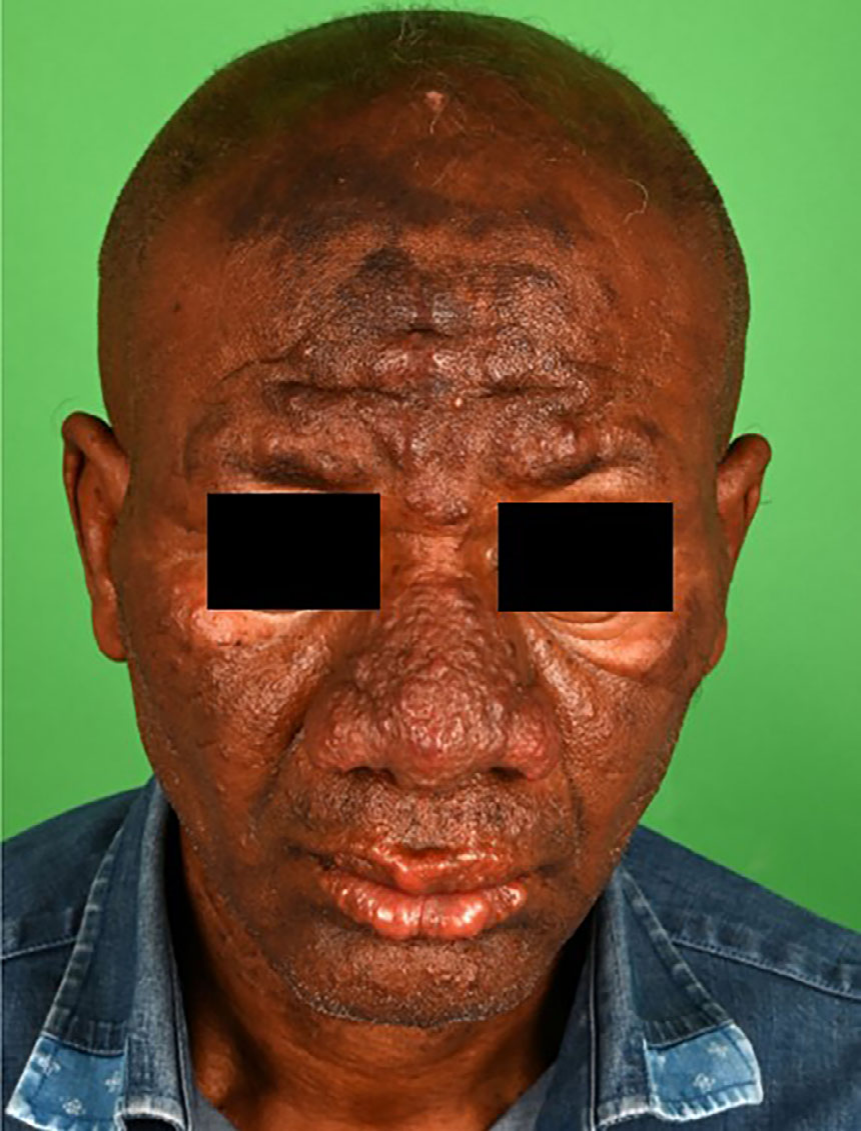
Hautbefund bei Erstvorstellung.

Es bestand der Verdacht auf eine multibazilläre, lepromatöse Lepra im Sinne einer typischen *facies leonina*. Die weiterführende Diagnostik wurde umgehend eingeleitet und der Verdachtsfall den zuständigen Gesundheitsbehörden gemeldet.

Mittels RLEP‐Real‐Time‐qPCR konnte *Mycobacterium leprae* aus Abstrichmaterial der Nasenschleimhaut sowie aus zwei Hautbiopsaten von der Stirn mit hoher Bakterienlast nachgewiesen werden (Nasenschleimhaut: ca. 35 000 Bakterien in 50 µL DNA‐Extrakt; Hautbiopsate: ca. 668 000 bzw. ca. 750 000 Bakterien in 50 µL DNA‐Extrakt). Serologisch fand sich ein hoher PGL‐1‐Titer. In der ergänzend durchgeführten konventionellen histopathologischen Untersuchung eines weiteren Hautbiopsats von der Stirn imponierte ein pandermales dichtes histiozytäres Infiltrat mit schaumigen Makrophagen (Virchow‐Zellen) ohne epidermale Veränderungen. Riesenzellen zeigten sich keine.

Mittels Fite‐Faraco‐Färbung konnten eindrucksvoll massenhaft intrazellulär, teils auch interstitiell gelegene Stäbchenbakterien nachgewiesen werden (Abbildung 2). Damit wurde die Verdachtsdiagnose Lepra zweifelsfrei bestätigt. Nebenbefundlich zeigte sich eine abgelaufene Hepatitis B, während sich serologisch keine Hinweise auf Hepatitis‐C‐ oder HIV‐Infektion ergaben.

**ABBILDUNG 2:**
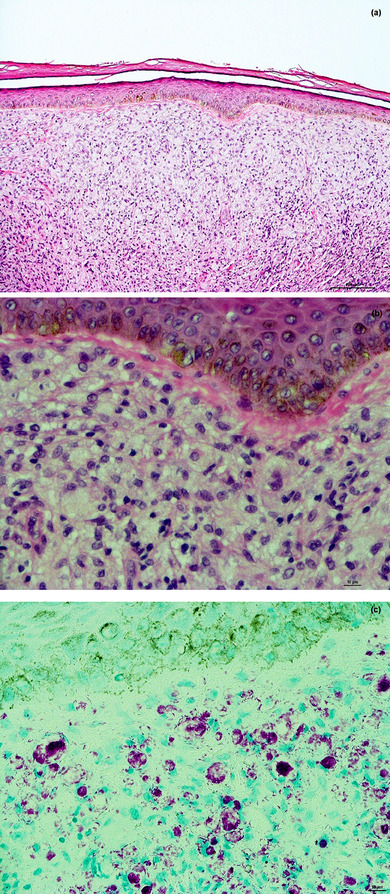
(a) Histopathologisches Bild eines Biopsats von der Stirn (Hämatoxylin‐Eosin‐Färbung, Originalvergrößerung x 100). Es zeigt sich ein pandermales histiozytäres Infiltrat ohne Riesenzellen. Die Makrophagen weisen ein großlumig schaumiges Zytoplasma auf (Virchow‐Zellen). Keine epidermale Beteiligung. (b) HE‐Färbung, x 400. (c) Fite‐Faraco‐Färbung (Originalvergrößerung x 400). Es imponierten massenhaft pink‐violett gefärbte, intrazellulär sowie interstitiell gelegene Stäbchenbakterien. Am oberen Bildrand ist ein Stück Epidermis miterfasst, welche unbeteiligt ist.

Nach Bestellung von Clofazimin über das Ausland konnte bereits wenige Tage nach der Erstvorstellung die von der Weltgesundheitsorganisation empfohlene einjährige Triple‐Antibiotika‐Therapie bestehend aus Clofazimin, Rifampicin und Dapson (Tabelle 1) eingeleitet werden.^1^ Erschwerend kam hinzu, dass der Aufenthaltsstatus und die Identität des Patienten sich zwischenzeitlich als unklar herausstellten, sodass zusammen mit den Behörden neben infektionshygienischen Fragen auch die Sicherstellung der Therapieadhärenz sowie nicht zuletzt die Kostenübernahme bei fehlender Krankenversicherung intensiv diskutiert werden mussten. Bislang erfolgt die Therapie ohne Nebenwirkungen oder Leprareaktionen. Die Auswirkungen der anamnestisch beschriebenen monoklonalen IgG‐Gammopathie unklarer Signifikanz bei unserem Fall bleiben abzuwarten. Hierzu sind in der Literatur bislang nur Einzelfallberichte beschrieben worden.^2^, ^3^

**TABELLE 1:** Therapieregime gemäß Empfehlung der WHO.^1^

Antibiotikum	Monatliche Einmaldosis	Tägliche Dosis
Rifampicin	600 mg p.o.	–
Clofazimin	300 mg p.o.	50 mg p.o.
Dapson	–	100 mg p.o.

Lepra ist eine der ältesten bekannten Krankheiten und begleitet die Menschheit bereits seit mehreren tausend Jahren. Untersuchungen zeigten, dass die DNA von *Mycobacterium leprae* über Jahrhunderte weitestgehend konserviert blieb.^4^ Genetische Faktoren, zum Beispiel im HLA‐Expressionsmuster, scheinen eine entscheidende Rolle zu spielen, ob Kontaktpersonen an Lepra erkranken.^5^ Aufgrund dieser Faktoren sowie der guten hygienischen Standards und eines gut ausgebauten Gesundheitssystems ist die Erkrankung in Europa selten geworden – während im Mittelalter noch sehr hohe Fallzahlen verzeichnet wurden. In Deutschland wurden zwischen 2001 und 2015 lediglich maximal fünf Fälle pro Jahr registriert.^6^ Bei den meisten Fällen handelt es sich um migrationsassoziierte Infektionen. Aufgrund mitunter eingeschränkter hygienischer Bedingungen und niedriger soziökonomischer Lebensstandards in diesem Kontext können direkte Übertragungswege jedoch nicht vollständig ausgeschlossen werden.^7^ Circa 95 % der Leprafälle treten mittlerweile im globalen Süden auf.^8^ Weltweit zeigen neu diagnostizierte Lepra‐Fälle im Zeitraum von 2006 bis 2016 laut Angaben der Weltgesundheitsorganisation eine rückläufige Tendenz.^1^

Da die Bakterien in der Nasenschleimhaut vorhanden sind, erfolgt die Übertragung wahrscheinlich aerogen sowie über Schmierinfektion.^9^ Die Inkubationszeit ist ungewöhnlich lange und kann 3 bis 7 Jahre betragen, in Einzelfällen sogar mehrere Jahrzehnte, wodurch Lepra die längste bekannte Inkubationszeit einer menschlichen Infektionserkrankung besitzt.^6^ *Mycobacterium leprae* vermehrt sich zunächst in Makrophagen der nasalen Schleimhaut über die es schließlich hämatogen zu Schwann‐Zellen der Myelinscheiden der peripheren Nerven gelangt.^6^ Makrophagen versuchen, den Erreger zu eliminieren. Aufgrund der Bildung lipidgefüllter Phagosomen werden die befallenen Makrophagen als Schaumzellen bezeichnet, die nach ihrem Erstbeschreiber Rudolf Virchow auch als Virchow‐Zellen bekannt sind.^6^ Je nach Immunitätslage des Infizierten manifestiert sich die Erkrankung in unterschiedlichen Stadien, die sich klinisch und histopathologisch deutlich unterscheiden. Hierzu zählen die beiden polarisierten Lepraformen: die *tuberkuloide (paucibazilläre) Lepra*, der eine Th1‐dominierte zelluläre Immunantwort zugrunde liegt, sowie die *lepromatöse (multibazilläre) Lepra*, bei der eine Th2‐Immunantwort überwiegt.^6^, ^10^ Zwischen diesen maximal polarisierten Lepraformen existieren Übergänge, welche als *Borderline‐Lepra* (Borderline‐tuberkuloide, Borderline‐Borderline und die Borderline‐lepromatöse Lepra) bezeichnet werden.^6^

Die klinische Verdachtsdiagnose kann bei der multibazillären Lepra durch PCR‐Untersuchungen von Gewebeproben und Nasenabstrichen meist schnell bestätigt werden.^11^, ^12^ Bei der paucibazillären Lepra ist die Sensitivität von PCR‐Untersuchungen geringer, sodass die Diagnosesicherung oft herausfordernd sein kann.^13^ Histopathologisch imponieren bei der tuberkuloiden Lepra meist Granulome aus Epitheloidzellen und Riesenzellen mit begleitendem lymphozytären Infiltrat. Bei der Borderline‐Lepra werden die Granulome mit zunehmender lepromatöser Polarisierung immer desorganisierter mit Abnahme von Epitheloidzellen, Riesenzellen und Lymphozyten, während Histiozyten zunehmen, bis schließlich das Vollbild der lepromatös polarisierten Lepra wie in unserem Fall zu finden ist.^14^

Dieser Fall zeigt eindrucksvoll das typische klinische und histologische Bild der in Europa äußerst selten zu beobachtenden multibazillären Lepra und verdeutlicht, dass aufgrund von Migrationsbewegungen nach Europa die Kenntnis über seltene Infektionserkrankungen für die korrekte und rasche Diagnosestellung und Therapieeinleitung entscheidend ist.

## DANKSAGUNG

Open access Veröffentlichung ermöglicht und organisiert durch Projekt DEAL.

## INTERESSENKONFLIKT

R.S. erhielt Referentenhonorare und/oder Reisekostenunterstützung von MedKom Akademie, KYOWA Kirin, Lilly, Eucerin, Unna Akademie, RG Gesellschaft für Information und Organisation, Sun Pharmaceutical Industries, Boehringer‐Ingelheim, Galderma, Pfizer und Novartis.
